# Changes in scalp potentials and spatial smoothing effects of inclusion of dura layer in human head models for EEG simulations

**DOI:** 10.3389/fneng.2014.00032

**Published:** 2014-08-05

**Authors:** Ceon Ramon, Paolo Garguilo, Egill A. Fridgeirsson, Jens Haueisen

**Affiliations:** ^1^Department of Electrical Engineering, University of WashingtonSeattle, WA, USA; ^2^Institute of Biomedical and Neural Engineering, Reykjavik UniversityReykjavik, Iceland; ^3^Department of Science, Landspitali University HospitalReykjavik, Iceland; ^4^Institute of Biomedical Engineering and Informatics, Technical University IlmenauIlmenau, Germany

**Keywords:** effect of dura on scalp potentials, spatial blurring due to dura, EEG and dura, EEG and cortex, EEG and ECoG models, EEG simulations, human head models for EEG

## Abstract

The dura layer which covers the brain is less conductive than the CSF (cerebrospinal fluid) and also more conductive than the skull bone. This could significantly influence the flow of volume currents from cortex to the scalp surface which will also change the magnitude and spatial profiles of scalp potentials. This was examined with a 3-D finite element method (FEM) model of an adult subject constructed from 192 segmented axial magnetic resonance (MR) slices with 256×256 pixel resolution. The voxel resolution was 1×1×1 mm. The model included the dura layer. In addition, other major tissues were also identified. The electrical conductivities of various tissues were obtained from the literature. The conductivities of dura and CSF were 0.001 S/m and 0.06 S/m, respectively. The electrical activity of the cortex was represented by 144,000 distributed dipolar sources with orientations normal to the local cortical surface. The dipolar intensity was in the range of 0.0–0.4 mA meter with a uniform random distribution. Scalp potentials were simulated for two head models with an adaptive finite element solver. One model had the dura layer and in the other model, dura layer was replaced with the CSF. Spatial contour plots of potentials on the cortical surface, dural surface and the scalp surface were made. With the inclusion of the dura layer, scalp potentials decrease by about 20%. The contours of gyri and sulci structures were visible in the spatial profiles of the cortical potentials which were smoothed out on the dural surface and were not visible on the scalp surface. These results suggest that dura layer should be included for an accurate modeling of scalp and cortical potentials.

## Introduction

The dura layer is sandwiched between the highly resistive skull bone above and less resistive CSF (cerebrospinal fluid) layer below. The conductivity of the dura layer is approximately 0.04 times the conductivity of the CSF (Oozeer et al., 2005). The inclusion of the dura layer in forward models will significantly reduce the volume currents flowing from cortical neurons to the skull bone. In turn, currents reaching the scalp surface will also be reduced which will decrease the scalp potentials. Additional factors influencing the forward solutions are the anisotropic properties of skull bone and 3-D profiles of CSF channels in-between the brain tissue.

The effects of skull bone and CSF on scalp potentials have been studies in detail by several authors (Ramon et al., 2006; Dannhauer et al., 2011; Lanfer et al., 2012). The dura layer, in general, has not been included in human head models for simulation of scalp potentials. Only recently some attention has been paid to examine the effect of the dura layer on scalp potentials (Slutzky et al., 2010; Kybartaite, 2012; Ramon, 2012). In our earlier work (Ramon, 2012), we used a nineteen-tissue finite element method (FEM) model of an adult male subject with randomly placed 120 dipoles in the upper (superior) portion of the cortex to examine the effect of the dura layer on scalp potentials. We found that inclusion of the dura layer reduced the scalp potentials by 29%. In another study, the dura layer was included in the model to simulate the scalp potentials due to three different dipolar sources in the parietal-occipital lobe (Kybartaite, 2012). No attempts were made to isolate and quantify the effect of the dura layer on scalp potentials. The study by Slutzky et al. (2010), examined the possible effects of dura on the spacing of surface electrodes for brain-computer interface applications. They performed the study by use of a six-tissue FEM model of the human head with a single dipolar source in the brain.

All of these studies have used few (1–120) dipoles which do not represent the spontaneous or cognitive electrical activity of the whole brain. Locations of few dipoles bias the spatial profiles of scalp potentials which will be different from the whole brain activity. The above described studies should be extended by representing the electrical activity of the brain with a large number of dipoles. This is the focus of the present work to examine spatial effects of the inclusion of the dura layer on scalp potentials from the electrical activity of the whole brain with a mm size cortical resolution.

## Methods

The scalp EEGs were simulated with a 1.0 mm resolution FEM model of a human head constructed from segmented magnetic resonance (MR) images. Our procedures for segmentation of MR images and FEM model constructions are described elsewhere (Ramon et al., 2006). Similar procedures were used and are described in brief. The T_1_ weighted MR images of 256×256 pixel resolution were collected with a 3-Tesla Siemens scanner at FS University, Jena, Germany. The subject was a 55-year-old female. The slice resolution was 1×1 mm and the contiguous slice thickness was 1.0 mm. This was a volumetric scan. With a 3-D image segmentation software, MIMICS,^1^ we identified 19 different tissue-types in the slices. These included: basal ganglion, blood, hard and soft skull, gray and white matter, cerebellum, corpus callosum, CSF, dura, eye, fat, muscle, salivary glands, scalp or skin, soft tissues, thalamus and internal air. Detailed structure of the nose, ear canals, eyes, and blood-filled sinus cavities were also included.

**Figure 1:**
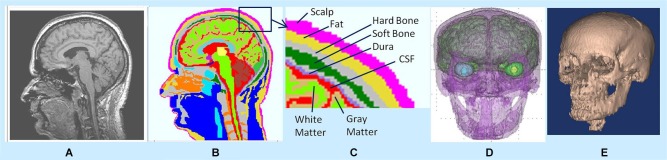
**(A)** Raw MR slice, **(B)** segmented slice, **(C)** details of tissue segmentations, **(D)** 3-D tissue surfaces, and **(E)** 3-D skull bone structure.

In MR images, the hard skull bone and CSF show up as dark pixels while the dura layer sandwiched between them shows up as bright pixels. Thus, bright pixels of dura help to correctly distinguish the boundary of the hard skull bone above the dura and the boundary of CSF layer below the dura. There are dark holes in MR images of the dura layer. However, with brightness/contrast enhancements and with other image processing techniques, such as, contour filling, edge detection etc., one can infer that the holes are actually part of the continuous dura layer. By use of these techniques and with the aid of a neuroanatomy atlas, we were able to segment the 3-D structure of dura layer surrounding the brain and the brain stem as one would expect to see in the anatomy of a person. Similar procedures were also used to segment the CSF and gray and white matter tissue boundaries. An example of 3-D segmentation is given in Figure 1. It shows a raw and segmented MR slice, 3-D tissue surfaces and 3-D skull bone structure.

A 3-D FEM head model was built from 192 segmented slices. The voxel resolution was 1×1×1 mm. The electrical conductivities of various tissues were obtained from the literature and are summarized in our previous work (Ramon et al., 2006). The conductivity of the dura matter is not well established and it was found to have a large range from 0.02 to 0.1 S/m (Oozeer et al., 2005). For our work, we used a midrange value of 0.06 S/m. The conductivity value of the corpus callosum was 0.12 S/m (Sekino et al., 2004). The conductivity and resistivity values of all tissues used in the FEM model are summarized in Table 1 and were compiled from the literature (Foster and Schwan, 1989; Gabriel et al., 1996a,b; Gabriel, 2005). Some of these values have been used earlier by us (Haueisen et al., 1997; Ramon et al., 2006; Ramon, 2012). The upper and lower bounds were usually set to 50% of the mean value. Only in the case of widely varying values in the literature, other bounds were chosen (Haueisen et al., 1997). These suggested lower and upper bounds represent a good approximation for all the resistivity values which can be found in the literature from human tissues and animal experiments.

**Table 1:** **Human head tissue resistivity and conductivity values**.

**Tissue**	**Mean Resistivity with lower/upper bounds (Ohm cm)**	**Mean Conductivity (S/cm)**
Air Internal	50,000 (50,000 to 100,000)	2E-5
Air External	100,000	1E-6
Basal Ganglia	700	1.42E-3
Blood	160 (80 to 240)	6.25E-3
Brain White Matter	700 (350 to 1,050)	1.42E-3
Brain Gray Matter	300 (150 to 450)	3.334E-3
Cerebellum	650 (325 to 975)	1.54E-3
Cerebrospinal Fluid	65 (32.5 to 97.5)	1.54E-2
Corpus Callosum	834	1.199E-3
Dura	1,667 (1,000 to 5,000)	6E-4
Eye	200 (100 to 400)	5E-3
Fat	2,500 (1,250 to 5,000)	4E-4
Muscle	1,000 (200 to 1,800)	1E-3
Salivary Glands	576	1.74E-3
Scalp and Skin	230 (115 to 345)	4.35E-3
Skull Hard Bone	16,000 (8,000 to 40,000)	6.25E-5
Skull Soft Bone	2,500 (1,250 to 3,750)	4E-4
Soft Tissue	500 (250 to 750)	2E-3
Thalamus	112	8.93E-3

*Mean values and lower and upper bounds of some of the tissues are included*.

The whole cortex had 144,000 voxels. The electrical activity in each cortical voxel was represented by a dipolar source which represented an averaged sum of volume currents of all neurons in one hypercolumn. Here our assumption is that a cortical voxel of 1×1×1 mm size can be treated as a hypercolumn. In general, a hypercolumn has about 80×10^3^ to 100×10^3^ neurons (Horton and Adams, 2005). The dipole intensity distribution in the whole cortex, i.e., in 144,000 voxels was in the range of 0.0–0.4 mA meter with a uniform random distribution. A total of ten runs was made with different uniform random distribution of dipolar intensities. The dipoles in the voxels were oriented normal to the local normal surface of the white and gray matter boundary with the orientation of the local normal vector pointing from white to gray matter.

**Figure 2:**
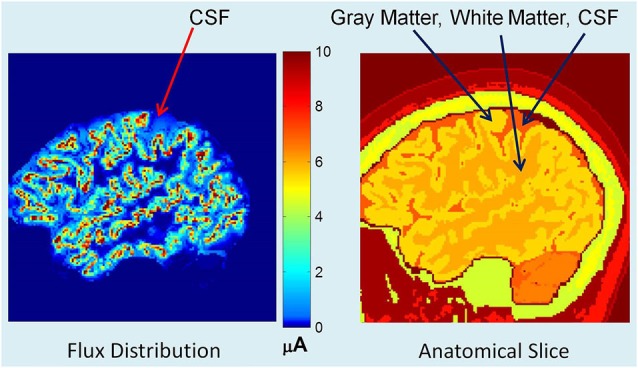
**(Left) Flux distribution, (right) corresponding segmented MR slice**. The red color in the left plot is the primary current source in each cortical voxel. The yellow and light blue colors show the spread of secondary volume currents in the gray matter. The medium blue color represents the spread of the volume currents in CSF.

Using an adaptive FEM solver, potentials and flux distributions in the whole head model were computed for a given dipole intensity distribution. An example of flux distribution is given in Figure 2. The left plot shows the primary and secondary volume current distribution. The corresponding anatomical slice (right plot) is also given with CSF, gray and white matter tissue boundaries identified. The red color in the left plot is the primary current source in each voxel representing the averaged electrical activity of a hypercolumn. The yellow and light blue colors show the spread of secondary volume currents in the gray and white matter. The medium and dark blue color represents the spread of the volume currents in CSF.

Scalp potentials were simulated for two head models. One model had the dura layer and in the other model, the dura layer was replaced with the CSF. The scalp potentials were extracted and referenced to a common average reference. Spatial contour plots of the scalp potentials above the eye-level were constructed.

Differences in the scalp potentials between two models were examined with Relative Difference Measure (RDM^*^) and magnification factor *(MAG)* (Meijs et al., 1989; Schimpf et al., 2002). The RDM^*^ is defined as:

(1) $$RDM*=\sqrt{{\sum_{j=1}^{m}\left[\frac{V_{k}^{RM}}{\sum_{k=1}^{m}{\left(V_{k}^{RM}\right)}^{2}}-\frac{V_{k}^{TM}}{\sum_{k=1}^{m}{\left(V_{k}^{TM}\right)}^{2}}\right]}^{2}}$$

where index, *j = 1:m*, runs over all the scalp points, $V_{k}^{RM}$ and $V_{k}^{TM}$ are the *k*^th^ scalp potentials for the reference model (RM) and the test model (TM). There were 48,640 scalp points above the eye level. Here we will use model with the dura layer as the reference model and the model in which dura layer was replaced with CSF as the test model. The *MAG* is defined as:

(2) $$MAG=\frac{\sqrt{\sum_{k=1}^{m}{\left(V_{k}^{TM}\right)}^{2}}}{\sqrt{\sum_{k=1}^{m}{\left(V_{k}^{RM}\right)}^{2}}}$$

The RDM^*^ is a measure of the difference in spatial profiles of two data sets and *MAG* is a measure of differences in the magnitude of the two data sets. If two data sets are the same, RDM^*^ will be zero and *MAG* will be unity.

## Results

### Dura effect on scalp potentials

**Figure 3:**
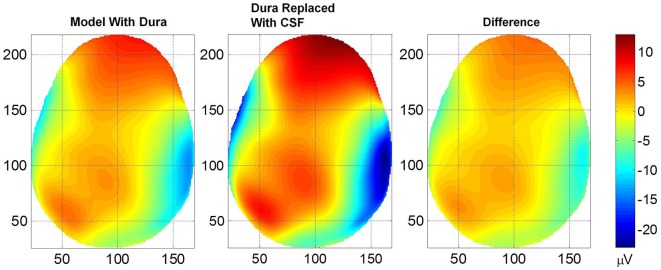
**Plot of scalp potentials**. (Left) model that contained dura layer, (middle) model in which the dura layer was replaced with CSF and (right) difference of the two plots. The color intensity scale is same for all three plots. In general, the inclusion of the dural layer in the FEM model severely reduces the magnitude of the scalp potentials as shown in the left plot.

The contour plots of scalp potentials above the eye level for the two models are given in Figure 3. In all plots, the nose (anterior) is on the top, left side of the subject is the left side of the plot, right side of the subject is the right side of the plot and bottom of the plot is the backside (posterior) of the head. The horizontal and vertical axes are in mm scale which is related to the pixel size of MR images and also to the 1×1×1 mm size FEM voxels. The left plot is for the model where the dura layer was included and the middle plot is for the model where the conductivity of the dura layer was set equal to the conductivity of CSF. The differences of scalp potentials for the two models are shown in the right plot. These plots show that the inclusion of the dura layer significantly reduces the scalp potentials. In the middle plot for the dura replaced with CSF, the peak value is 12.5 μV at the top portion of the plot. At the same location in the left plot the value is 7.5 μV. Thus, at this location, the inclusion of the dura layer has reduced the amplitude of the scalp potential by 40%.

**Figure 4:**
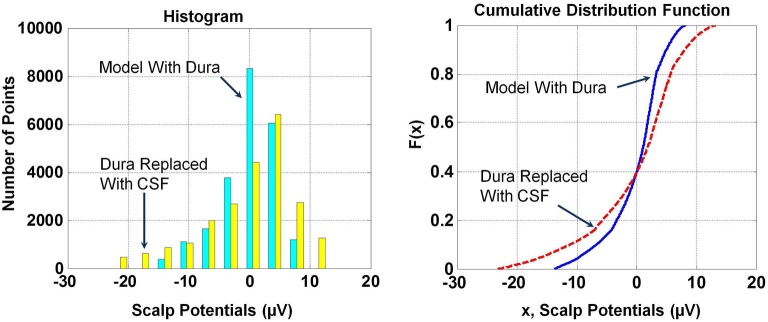
**Histograms and cumulative distribution functions**, ***F(x)*, of scalp potentials for the model with dura and the model where dura was replaced with CSF**. Histograms and *F(x)* are different for scalp potentials derived from the two models.

The RDM^*^ and *MAG* values of scalp potentials for the two models are 0.05 and 1.66, respectively, suggesting that the spatial profiles of two plots are similar but their magnitudes are significantly different. The histogram and Cumulative Distribution Function (CDF), *F(x)*, of scalp potentials, *x*, for two models are given in Figure 4. These are derived from scalp potentials given in Figure 3. The histogram and CDF values are different for the two models. The Kolmogorov–Smirnov test (K–S test) was also performed on the *F(x)* of two models and the null hypothesis was rejected suggesting that *F(x)* of scalp potentials for the two models are different.

Ten trial runs were made with different uniform distribution of cortical dipole intensities in the range of 0.0–0.4 mA meter. The spatial plots of scalp potentials were different as compared with Figure 3 for each trial run. The mean and standard deviation of RDM^*^ and *MAG* were: 0.057 ± 0.0003 (*n* = 10) and 1.68 ± 0.0008 (*n* = 10), respectively. Overall, from these results one can conclude that the inclusion of the dura layer significantly changes the magnitude of scalp potentials and slightly changes the spatial profiles.

### Spatial profiles on different tissue surfaces

**Figure 5:**
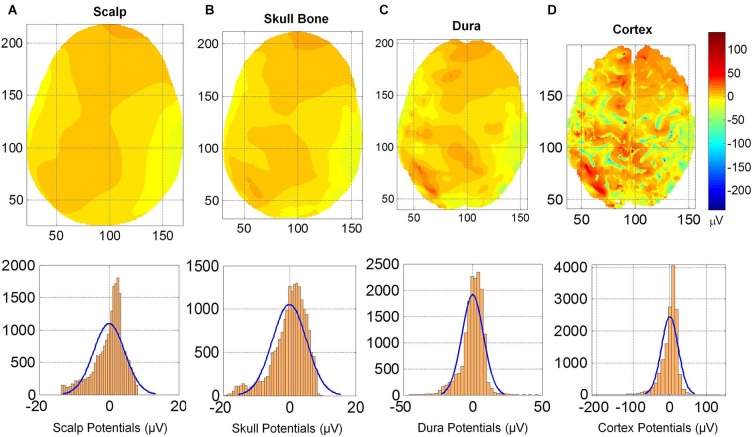
**(Top row) Scalp potentials on (A) scalp, (B) upper skull surface, (C) upper dura surface and (D) the cortical surface.** (Bottom row) Histograms and normal density functions for the potentials on all four surfaces. Progressive blurring of spatial profiles is visible as one moves from the cortical surface toward the scalp.

The spatial profiles of potentials on the cortical surface, outer surface of the dura, outer surface of skull bone and on the scalp are given in Figure 5. The spatial profiles are given in the top row and the histogram of potentials are given in the bottom row. The anatomical structures of the gyri and sulci are clearly visible in spatial profiles of cortical potentials (Figure 5D) which are absent in the spatial profiles of dura potentials (Figure 5C). One can observe a progressive blurring of spatial details as one moves from cortical surface to dura, to skull bone and finally to the scalp surface. There are large number of well-defined contour shapes on the dura surface which are blurred out to fewer contours on the outer skull bone surface. There are only two well defined top and bottom contours on the outer skull surface which are further blurred out to one large contour on the scalp surface.

This blurring of spatial contours on different tissue surfaces is also reflected in the histogram of potentials given in the bottom row of Figure 5. The histogram of cortical potentials has a large distribution in the ±100 μV range and lesser number of points outside that range. On the outer dura surface, the distribution is in the ±50 μV. On the outer skull bone surface, the histogram distribution is in the range of +10 to −20 μV and on the scalp it is in the range of +8 to −15 μV range. These ranges for histograms are expected because the magnitude of potentials decreases as one moves from cortical surface to the scalp. Mean and standard deviation values of the potentials are: (1) for the cortex: 0.0 ± 22.8 μV; (2) for the outer dura surface: 0.0 ± 7.71 μV; (3) for the outer skull surface: 0.0 ± 5.16 μV; and (4) for the scalp: 0.0 ± 4.47 μV. The mean values are zero because the potentials were referenced to the common average of the potentials for each case. The standard deviation gives a spread of the potentials on the tissue surface. It is largest on the cortical surface. It is smaller for the scalp and the outer skull bone surface.

**Figure 6:**
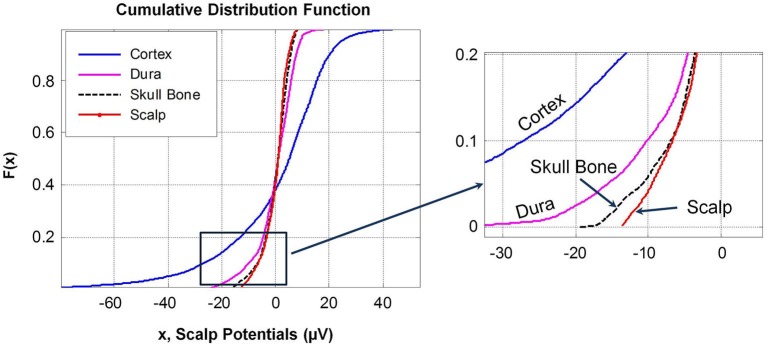
**Cumulative distribution functions, *F(x)*, of potentials on different tissue surfaces and the outer skull bone surface**. A magnified view of the lower end of *F(x)* is given in the right plot. *F(x)* of dura and cortex potentials are different while *F(x)* of scalp and outer skull bone potentials are similar with noticeable differences at the upper and lower tail ends of the distribution curves.

Cumulative distribution functions, *F(x)*, of potentials on different surfaces are given in Figure 6. The *F(x)* of potentials on the scalp and the outer surface of skull bone are similar because the drop in potentials is small in the muscle and fat layers which are located between the scalp and the skull bone structure. This is also reflected in the similarity of the histograms of potentials in the range of +8 to −15 μV for the scalp and the outer surface of skull bone. The *F(x)* distribution of potentials on the cortical surface and on the outer surface of the dura layer are different. This was also confirmed by the K–S test.

### Potentials on the inner and outer dura surface

**Figure 7:**
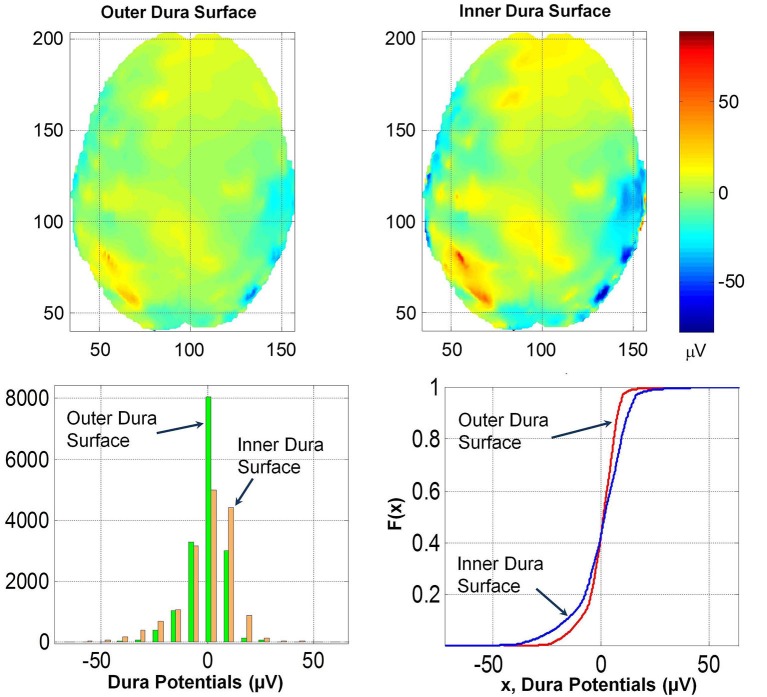
**(Top row) Contour profiles of potentials on the outer and inner dura surfaces**. (Bottom left) Histogram distribution and (bottom right) cumulative distribution functions, *F(x)*. Contour profiles have only minor differences which are also reflected in similarities of histograms and also in *F(x)*.

To further quantify the effect of dura on potentials, the spatial plot of the potentials on the outer and inner dura surfaces were constructed. These are given in Figure 7. The inner dura surface is defined as closer to the cortical surface and the outer dura surface is defined as closer to the inner skull bone surface. The RDM^*^ and *MAG* values of potentials for the two dura surfaces are 0.32 and 1.63, respectively. This suggests that the spatial profiles on the inner and outer dura surface are significantly different. This is also reflected in the histogram and *F(x)* distribution of potentials on the two surfaces. These distributions are also given in the Figure 7. The K–S test was performed on *F(x)* of potentials on the inner and outer dura surface and it was found that the two distributions are different.

### Potentials on inner and outer skull surface

**Figure 8:**
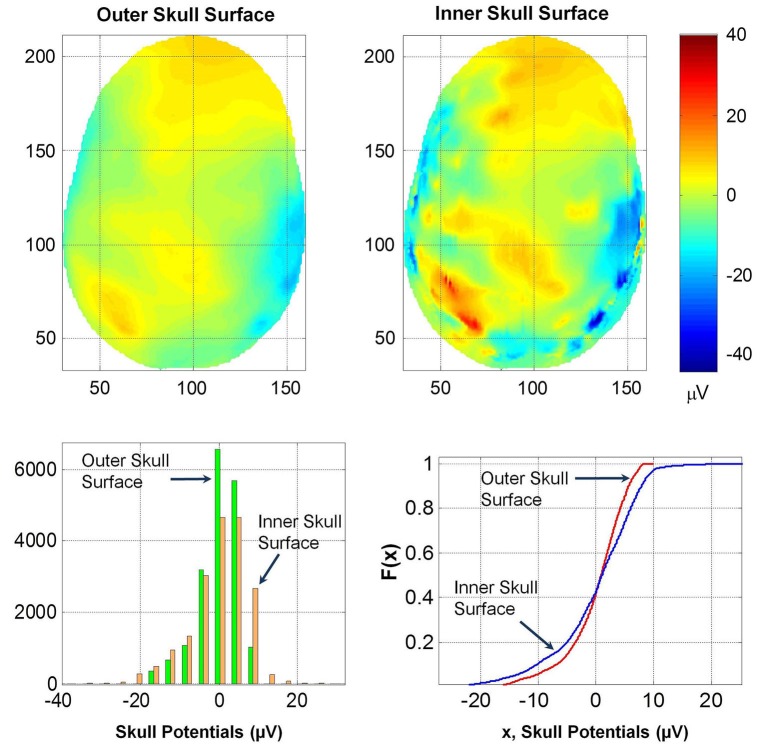
**(Top row) Contour profiles of potentials on the inner and outer skull surfaces**. (Bottom left) Histogram distributions and (bottom right) cumulative distribution functions, *F(x)*. Notice that contours are blurred on the outer skull surface.

The spatial profiles of potentials on the outer and inner skull surfaces are given in Figure 8. Several contour details are visible on the inner skull surface which are blurred on the outer skull surface. The RDM^*^ and *MAG* values of potentials on the outer and inner skull surfaces are 0.5 and 1.44, respectively. This also suggests that the thickness of skull bone changes the spatial profiles and also the magnitude of the potentials. This likely will be due to anisotropy effects of the soft skull bone and the high resistivity of the hard and soft skull bone. The histogram and *F(x)* distributions of potentials on the two surfaces are also different. The K–S test also confirmed that the two *F(x)* distributions are different.

## Discussions

These results suggest that the dura layer reduces the magnitude of scalp potentials significantly and should be included in human head models for accurate simulation of scalp EEGs. This will help to accurately relate the cortical volume currents to scalp EEGs. We have also examined how thicknesses of the dura and scalp both influence the spatial profiles and also the magnitude of the potentials. Please refer to Figures 7, 8. Thus, in forward EEG simulations it is necessary to include detailed 3-D structures of soft and hard skull bones and the dura. This will also help in reducing the blurring effects while reconstructing the cortical sources from the scalp EEG data. Reduction in blurring due to the skull has been proposed by use of FEM models of the head that include 3-D structure of the skull bone (Le and Gevins, 1993; Gevins et al., 1999). This blurring can be further reduced in reconstruction of the cortical sources from scalp EEG data by including 3-D detailed structures of the soft and hard skull bone and the dura layer in FEM models of the head. The inclusion of the dura layer will also help in better design of brain-computer interface for medical applications.

The segmentation of the dura layer is difficult but can be done by use of image enhancement techniques and with the aid of a neuroanatomy atlas. In an adult brain dura layer is approximately 0.8–0.9 mm thick (Genina et al., 2005). Thus, in MR images with 1.0 mm resolution there is a slight overestimation of dura volume which is an error due to the limits of 3.0 Tesla MR imaging systems. A sub-millimeter pixel resolution can be achieved with higher field strength (>4 Tesla) MR imaging systems which will help in better imaging and segmentation of the dura layer. This slight overestimation of the thickness of the dura layer should not significantly compromise our reported results.

The skull bone is poorly conductive and the dura layer is added next to the inner surface of the hard skull bone which is moderately more conductive than the hard skull bone. Thus, it is a possibility that the variations in hard skull bone conductivity could overshadow the effects related to the inclusion of the dura layer in head models. The scalp potentials given in Figure 3 were computed with the model having a mean hard skull bone conductivity of 6.25E-5 S/cm (resistivity of 16,000 Ohm cm). The RDM^*^ and *MAG* values were 0.05 and 1.66, respectively for the two models with and without the dura layer. We recomputed scalp potentials by varying the hard scull bone conductivity by ±50% from the mean value. For the hard bone skull conductivity of 4.167E-5 S/cm (resistivity of 24,000 Ohm cm), the RDM^*^ and *MAG* values were 0.067 and 1.29, respectively. Similarly, for the hard bone skull conductivity of 1.25E-4 S/cm (resistivity of 8000 Ohm cm), the RDM^*^ and *MAG* values were 0.06 and 1.39, respectively. For two plots to be similar, RDM^*^ should be zero and *MAG* should be unity. Thus, these results suggest that by varying the hard skull bone conductivity, the changes in scalp potentials due to the inclusion of the dura layer are not totally suppressed. The *MAG* values of 1.29 or 1.35 indicate that there were differences in the magnitude of scalp potentials of two models with and without the dura layer. Similarly, RDM^*^ values of 0.067 or 0.06 indicate that the spatial profiles of two models, with and without the dura layer, are also slightly different. More detailed studies are needed to examine how conductivity variations of various tissues, such as, skull bone, CSF, gray and white matter affect scalp potentials when the dura layer is included in head models.

In summary, even with above described limitations, our results show that dura layer plays an important role and should be included in forward simulations of EEGs under normal and abnormal conditions.

## Conflict of interest statement

The authors declare that the research was conducted in the absence of any commercial or financial relationships that could be construed as a potential conflict of interest.

## Footnotes

^1^http://biomedical.materialise.com/mimics

## References

[B1] Dannhauer M. Lanfer B. Wolters C. H. Knösche T. R. (2011). Modeling of the human skull in EEG source analysis. Hum. Brain Mapp. 32, 1383–1399 10.1002/hbm.21114 20690140 PMC6869856

[B2] Foster K. R. Schwan H. P. (1989). Dielectric properties of tissues and biological materials: a critical review. Crit. Rev. Biomed. Eng. 17, 25–104 2651001

[B3] Gabriel C. (2005). Dielectric properties of biological tissue: variation with age. Bioelectromagnetics 26(Suppl. 7), S12–S18 10.1002/bem.20147 16142779

[B4] Gabriel C. Gabriel S. Corthout E. (1996a). The dielectric properties of biological tissues: I. Literature survey. Phys. Med. Biol. 41, 2231–2249 10.1088/0031-9155/41/11/001 8938024

[B5] Gabriel S. Lau R. W. Gabriel C. (1996b). The dielectric properties of biological tissues: II. Measurements in the frequency range 10 Hz to 20 GHz. Phys. Med. Biol. 41, 2251–2269 10.1088/0031-9155/41/11/002 8938025

[B6] Genina É. A. Bashkatov A. N. Kochubey V. I. Tuchin V. V. (2005). Optical clearing of human dura mater. Opt. Spectrosc. 98, 470–476 10.1134/1.1890530

[B7] Gevins A. Le J. Leong H. McEvoy L. K. Smith M. E. (1999). Deblurring. J. Clin. Neurophysiol. 16, 204–213 10.1097/00004691-199905000-00002 10426404

[B18] Haueisen J. Ramon C. Eiselt M. Brauer H. Nowak H. (1997). Influence of tissue resistivities on neuromagnetic fields and electric potentials studied with a finite element model of the head. IEEE Trans. Biomed. Eng. 44, 727–735 10.1109/10.605429 9254986

[B19] Horton C. J. Adams D. L. (2005). The cortical column: a structure without a function. Philos. Trans. R. Soc. Lond. B Biol. Sci. 360, 837–862 10.1098/rstb.2005.1623 15937015 PMC1569491

[B8] Kybartaite A. (2012). Computational representation of a realistic head and brain volume conductor model: electroencephalography simulation and visualization study. Int. J. Numer. Method. Biomed. Eng. 28, 1144–1155 10.1002/cnm.2483 23109383

[B9] Lanfer B. Scherg M. Dannhauer M. Knösche T. R. Burger M. Wolters C. H. (2012). Influences of skull segmentation inaccuracies on EEG source analysis. Neuroimage 62, 418–431 10.1016/j.neuroimage.2012.05.006 22584227

[B10] Le J. Gevins A. (1993). Method to reduce blur distortion from EEG’s using a realistic head model. IEEE Trans. Biomed. Eng. 40, 517–528 10.1109/10.237671 8262533

[B11] Meijs J. W. Weier O. W. Peters M. J. van Oosterom A. (1989). On the numerical accuracy of the boundary element method. IEEE Trans. Biomed. Eng. 36, 1038–1049 10.1109/10.40805 2793196

[B12] Oozeer M. Veraart C. Legat V. Delbeke J. (2005). Simulation of intra-orbital optic nerve electrical stimulation. Med. Biol. Eng. Comput. 43, 608–617 10.1007/bf02351034 16411633

[B13] Ramon C. (2012). Effect of dura layer on scalp EEG simulations. Int. J. Bioelectromagn. 14, 27–28

[B14] Ramon C. Schimpf P. Haueisen J. (2006). Influence of head models on EEG simulations and inverse source localizations. Biomed. Eng. Online 5:10 10.1186/1475-925X-5-10 16466570 PMC1389789

[B15] Schimpf P. Ramon C. Haueisen J. (2002). Dipoles models for the EEG and MEG. IEEE Trans. Biomed. Eng. 49, 409–418 10.1109/10.995679 12002172

[B16] Sekino M. Inoue Y. Ueno S. (2004). Magnetic resonance imaging of mean values and anisotropy of electrical conductivity in the human brain. Neurol. Clin. Neurophysiol. 2004:55 16012645

[B17] Slutzky M. W. Jordan L. R. Krieg T. Chen M. Mogul D. J. Miller L. E. (2010). Optimal spacing of surface electrode arrays for brain-machine interface applications. J. Neural Eng. 7:26004 10.1088/1741-2560/7/2/026004 20197598 PMC2844916

